# A comparative exploration of mRNA capping enzymes

**DOI:** 10.1016/j.biotno.2024.11.005

**Published:** 2024-11-20

**Authors:** Yiming Wang, Xiaoxue Wang, Wenchao Li, Xinjie Chen, Yuan Lu

**Affiliations:** a Department of Chemical Engineering, Tsinghua University, Beijing, 100084, China; b Key Laboratory of Industrial Biocatalysis, Ministry of Education, Tsinghua University, Beijing, 100084, China

**Keywords:** mRNA capping enzyme, Heterologous expression, Soluble tag, Transcription

## Abstract

With the wide application of messenger RNA (mRNA) technology in medicine and vaccine fields, higher requirements are put forward for mRNA expression efficiency *in vivo*. Since the 5′ cap structure can spatially protect mRNA from exonuclease degradation and enhance the initiation of translation reactions, *in vitro* mRNA caps are a promising option to improve the efficiency of mRNA expression *in vivo*. In order to obtain more efficient mRNA capping enzymes, seven mRNA capping enzymes from different viral sources were explored in this study. Eukaryotic and prokaryotic cells were used for the heterologous expression of the cap enzymes, and *Escherichia coli* was identified as the most suitable host cell for heterologous expression. In addition, in order to improve the solubility of the capping enzyme, four kinds of soluble labels were screened, among which maltose-binding protein had the best effect and the widest applicability. The mRNA was then transfected into the human cells, and the highest transfection efficiency was achieved using the bluetongue virus capping enzyme. Its effect was 38 % higher than that of the previously widely used vaccinia virus capping enzyme. This work will promote the development of mRNA technology and expand its application space.

## 1 Introduction

Messenger RNA (mRNA) is a single-stranded ribonucleic acid transcribed from a DNA strand as a template and can guide protein synthesis.^1^^,^^2^ The success of mRNA expression in animals confirmed the feasibility of mRNA therapy.^3^ Later, the discovery of pseudouridine improved mRNA stability and opened the prelude of mRNA entering the clinic.^4^ In theory, mRNA can express any protein and prevent many diseases. Compared with DNA therapy, mRNA does not need to enter the nucleus and has higher safety and transfection efficiency. Compared with protein therapy, mRNA drugs are simple to manufacture, have low cost, and have better efficacy. mRNA vaccine technology won the 2023 Nobel Prize in Physiology and Medicine. Due to these characteristics, the field of mRNA therapy has been paid more and more attention. It has been widely used in the fields of tumor vaccine,^5^ infectious disease vaccine,^6^ protein replacement therapy,^7^^,^^8^ and rare disease therapy.^9^

**Fig. 1:**
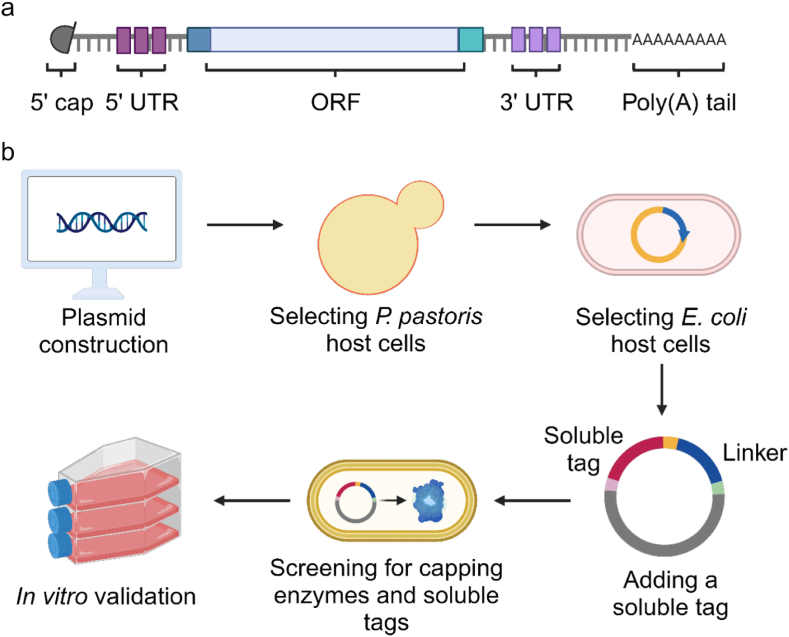
**The 5′ end modification strategy of mRNA**. (a) Composition of mRNA. (b) Optimization strategy of mRNA capping enzyme.

The mature mRNA structure of eukaryotic cells usually includes a 5′ cap structure, 5′-untranslated region (5′-UTR), open reading frame (ORF), 3′-untranslated region (3′-UTR), and poly(A) tail (Fig. 1). To ensure the stable expression of mRNA, it is necessary to stimulate the mechanism of eukaryotic mRNA synthesis to complete mRNA transcription *in vitro*.^10^^,^^11^ The selection of mRNA sequence, structure optimization, modification, and lipid nanoparticle (LNP) delivery system is very important.^12^, ^13^, ^14^, ^15^ The 5′-end cap reaction is an essential modification process, and the cap structure formed helps mRNA escape the innate immune response of cells and achieve effective protein translation *in vivo*.^16^

Currently, there are two general methods for producing capped mRNA *in vitro*. One is capping with cap structure analogs. The other is capping with mRNA capping enzymes. The analog method is realized by directly adding cap structure analogs in high concentrations to the *in vitro* transcription reaction, which can significantly reduce the difficulty of mRNA modification.^17^ However, part of cap structure analogs will reverse their localization to the 3′ end of mRNAs. The enzyme method uses mRNA capping enzyme to catalyze the mRNA capping reaction after the *in vitro* transcription reaction. The enzymatic capping reaction was completed within 1 h, the capping efficiency was close to 100 %, and all cap structures were added in the correct direction. The analog method is simple but less efficient and accurate than the enzyme method. Therefore, the enzyme method is considered a more promising method for mRNA capping *in vitro*. Currently, the vaccinia virus capping enzyme (VCE) is the main commercial capping enzyme.^18^^,^^19^ However, the activity of current commercially available VCE is still limited. It is essential to find out more efficient mRNA capping enzymes.

In this study, to improve the efficiency of the mRNA capping reaction, mRNA capping enzymes of different viral origins were selected and screened. Heterologous expression of mRNA capping enzymes from different viral origins was performed based on different protein expression systems, such as *Pichia pastoris* X33 and *Escherichia coli* BL21 (DE3). On this basis, a soluble tag was introduced to improve the solubility of the mRNA capping enzymes. Then, the capped mRNAs were transfected to cells to screen out the mRNA-capping enzyme with the highest translational ability. This work would promote the development of the mRNA technique and expand its application range.

## 2 Materials and methods

### 2.1 Enzymes, plasmids, and DNA templates

Vaccinia capping enzyme (M2080) was purchased from New England Biolabs. For protein expression in yeast, pPICZalpha A was used as a plasmid vector (Fig. S1). For protein expression in *E. coli*, pET-21a (+) was used as a plasmid vector (Fig. S2). DNA templates were all PCR-linearized using KOD FX kits (TOYOBO). Amino acid sequences of mRNA capping enzymes from different viral sources were shown in Table S2. The nucleic acid and amino acid sequences of the soluble tags were shown in Table S3.

### 2.2 *In vitro* transcription and purification

The 25 μL reaction system included 5 μL 5′ Transcription Buffer, 4 μL 10 mM rNTPs Mix, 2.5 μL 100 mM DTT, 1 μg Template DNA, 2 μL T7 RNA Polymerase, and DEPC-treated H_2_O. The components of 5′ Transcription Buffer included: 50 mM NaCl, 40 mM MgCl_2_, 10 mM spermidine, and 400 mM Tris-HCl (pH 8.0). The detailed operations of *in vitro* transcription were as follows. Thawed the components except for T7 RNA Polymerase on ice. Mixed all components in an RNase-free environment. The scale of the reaction system could be appropriately increased according to the experimental requirements. Incubated at 37 °C for 2 h. If the target mRNA was less than 300 nt, the reaction time could be extended to 4 h or 16 h. After incubation, 1 μL DNase I was added to the 25 μL reaction system to degrade Template DNA. Incubated at 37 °C for 10–15 min. Then mRNA was purified by MEGAclear™ Transcription Clean-Up Kit (ThermoFisher). The concentration was measured by Nanodrop. Stored in a −80 °C refrigerator for future use.

### 2.3 *In vitro* mRNA capping reaction

The 20 μL mRNA capping reaction system included 2 μL 10 × Capping Buffer, 1 μL 10 mM GTP, 1 μL 2 mM SAM, 10 μg mRNA, 1 μL capping enzyme, and DEPC-treated H_2_O. The components of the 10 × Capping Buffer included 5 mM KCl, 1 mM MgCl_2_, 1 mM DTT, and 40 mM Tris-HCl (pH 8.0). The detailed operations of *in vitro* mRNA capping reaction were as follows. Thawed the components except for the capping enzyme on ice. Meanwhile, the mRNA sample was heated at 65 °C for 5 min. After heating, the mRNA sample was placed on ice for 5 min. Mixed all the components in an RNase-free environment. Incubated at 37 °C for 30 min. If the target mRNA was shorter than 300 nucleotides, the reaction time could be extended to 2 h. Then mRNA was purified by MEGAclear™ Transcription Clean-Up Kit (ThermoFisher). The concentration was measured by Nanodrop. Stored in a −80 °C refrigerator for future use.

### 2.4 Cell culture, transfection, and flow cytometry

HEK293T was cultured at 37 °C, and 5 % CO_2_ in Dulbecco's Modified Eagle's Medium (Gibco) supplemented with 10 % fetal bovine serum. Lipofectamine™ MessengerMAX™ Transfection Reagent (Thermofisher) was used for transfection. Fluorescence was detected for 10,000 events on a BD FACSCelesta flow cytometer (BD Biosciences). Data was analyzed in Flowjo (Flowjo LLC).

## 3 Results

### 3.1 Screening high expression mRNA capping enzymes

Compared with mRNA-capping enzymes from eukaryotic cells, viral-derived mRNA-capping enzymes had higher functional integration.^20^ Therefore, the current screening direction of mRNA capping enzymes was mainly for viral-derived mRNA capping enzymes. Based on these properties, viral-derived mRNA capping enzymes with similar activities were screened from various protein databases and enzyme databases (Table 1). These include pNP868R (from African swine fever virus), VP4 (from bluetongue virus), P5 (from rice dwarf virus), VP3 (from rotavirus), VCE (from cowpox virus), CHL (from chlorella virus), and FCE (from faustovirus).

**Table 1:** mRNA capping enzymes from different viral sources.

Source	Abbreviation	PDB ID	NCBI ID
African swine fever virus	pNP868R	7D8U	QZK26801.1
Bluetongue virus	VP4	2JHA	ASV51737.1
Rice dwarf virus	P5	5X70	5X6X_D
Rotavirus	VP3	4YE2	ATI15038.1
Cowpox virus	VCE	–	ARB50340.1
Chlorella virus	CHL	1CKO	Q84424.1
Faustovirus	FCE	–	QJX72631.1

In theory, numerous enzymes can be used for screening. Considering the feasibility of the research, we first selected enzymes that have a certain specific foundation in previous studies. This study focuses on verifying whether positive screening can indeed be obtained from enzyme species from other virus sources. In the future, two strategies can be adopted. One is screening more effective enzymes from databases. The other one is the rational design and mutation of the current effective enzyme.

### 3.2 Expression of mRNA capping enzymes based on *P. pastoris* X33

At first, yeast was selected for the expression of mRNA capping enzymes. Yeast has a well-developed mechanism for regulating gene expression and then can modify the translated protein, which can be secreted and expressed, and the purification process is simple and safe.^21^ Among them, *P. pastoris* X33 could express higher protein levels and have a stronger ability to modify proteins. Therefore, *P. pastoris* X33 strain was used as the host cell for the heterologous enzyme expression, and pPICZalpha A was used as a plasmid vector.

**Fig. 2:**
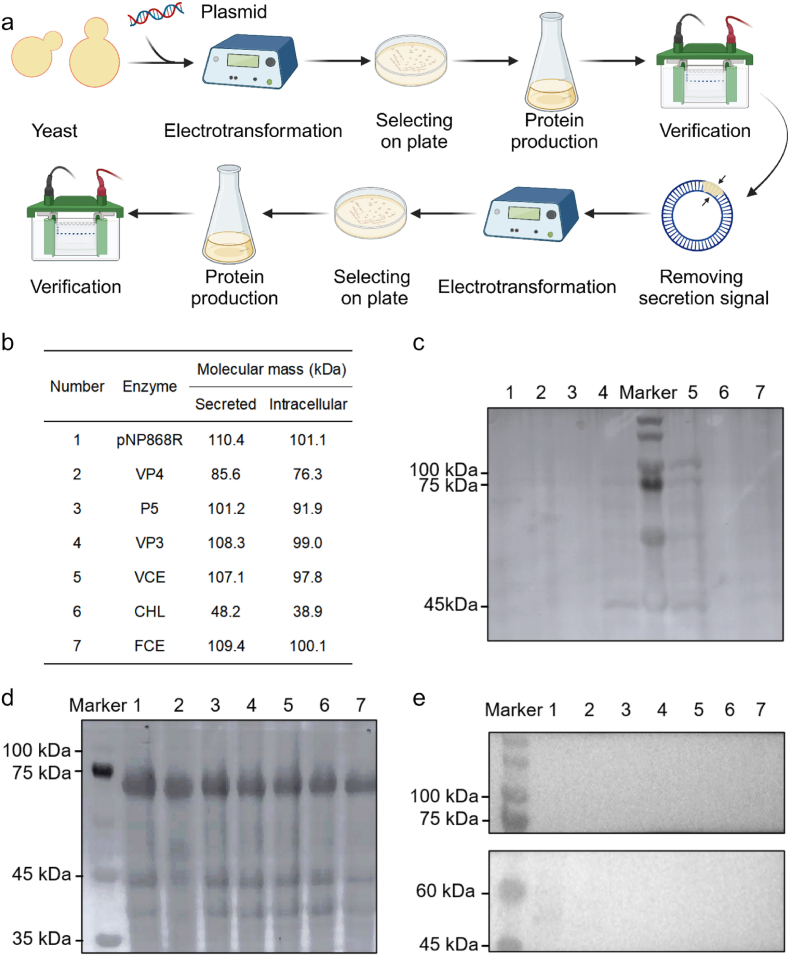
**Expression of mRNA capping enzymes in *P. pastoris* X33**. (a) The strategy of the secreted expression of mRNA capping enzymes in *P. pastoris* X33. (b) Different mRNA capping enzymes and their molecular mass. (c) SDS-PAGE results of the secreted expression of mRNA capping enzymes in *P. pastoris* X33. (d) Western blot results of the secreted expression of mRNA capping enzymes in *P. pastoris* X33. (e) Western blot results of intracellular expression of mRNA capping enzymes in *P. pastoris* X33.

The expression of mRNA capping enzymes in *P. pastoris* was detected and analyzed. First, the obtained extracellular samples were verified by SDS-PAGE, and no obvious target protein bands were observed in SDS-PAGE gel. Even when all the proteins were concentrated using ultrafiltration tubes, there were still no bands in the SDS-PAGE gel (Fig. 2c). This indicated that the enzyme was expressed at a low level or not expressed in *P. pastoris* X33. Further, the extracellular samples were characterized by Western blot, which still did not show the target protein bands (Fig. 2d). This indicated that the exogenous enzyme was not secreted from *P. pastoris* X33. The failure of initial experiments in yeast might be due to the fact that the tested enzymes were not suitable for secretory expression and could not be successfully secreted from yeast cells across the cell membrane. To solve this problem, two approaches have been taken. One was to remove the plasmid's signaling peptide, and the other was to use another expression system that was generally more efficient and less costly.

To explore whether mRNA capping enzymes were expressed in *P. pastoris*, the secretion signal of the target plasmid was removed. The resulting intracellular sample was still verified by Western blot (Fig. 2e). No protein bands of the target enzyme were found in the gel. The results showed that the mRNA capping enzyme was not successfully expressed in *P. pastoris* X33 cells after removing the secretion signal. This result might be because the codon of the foreign gene did not match the preference of *P. pastoris* X33. In the future, codon optimization and enzyme expression exploration could be performed in other yeast strains. Due to the high time-consuming process of genetic engineering in eukaryotic yeast, to quickly promote this study, the prokaryotic host was investigated for heterologous expression of the mRNA capping enzyme next.

### 3.3 Expression of mRNA capping enzyme based on *E. coli* BL21 (DE3)

*E. coli* is one of the most widely used engineering bacteria and is often used to express heterologous proteins. *E. coli* had a clear genetic background and mild culture conditions and could be easily introduced into exogenous plasmids for amplification and expression. Therefore, *E. coli* was selected for the expression of mRNA capping enzymes. *E. coli* BL21, currently the most widely used host cell, lacks Lon and OmpT proteases, which increase the production of recombinant proteins and are commonly used to express non-toxic proteins. Strain *E. coli* BL21 (DE3) is a derivative of strain BL21. The *E. coli* BL21 (DE3) strain integrates the gene encoding the T7 RNA polymerase based on the *E. coli* BL21 strain, so it was used to express the plasmid vector containing the T7 promoter.

**Fig. 3:**
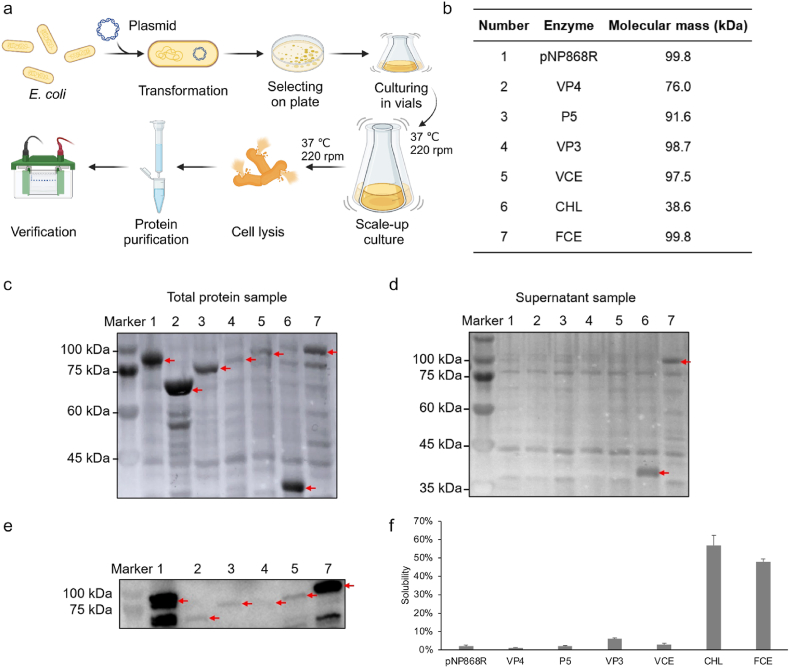
**Expression of mRNA capping enzymes in *E. coli* BL21(DE3)**. (a) Diagram of mRNA capping enzyme expression in *E. coli* BL21 (DE3). (b) Different mRNA capping enzymes and their molecular mass. (c) SDS-PAGE results of the secreted expression of mRNA capping enzymes in *E. coli* BL21 (DE3). (d) SDS-PAGE results of the secreted expression of mRNA capping enzymes in *E. coli* BL21 (DE3). (e) Western blot results of the secreted expression of mRNA capping enzymes in *E. coli* BL21 (DE3). (f) Solubility analysis of different capping enzymes.

The recombinant protein expression in *E. coli* BL21 (DE3) was detected and analyzed. The total protein sample and supernatant sample were analyzed using SDS-PAGE. The gel results of the total protein sample showed that all mRNA capping enzymes were successfully expressed in *E. coli* BL21 (DE3) (Fig. 3c). Among them, the expression levels of pNP868R, VP4, P5, and CHL were higher in *E. coli* BL21 (DE3). In contrast, only the bands of CHL were found among the supernatant samples, and the remaining six enzymes had no obvious protein bands (Fig. 3d). The results indicated that only CHL had good solubility, while the rest of the enzymes had low solubility. This result might be due to the smaller molecular weight of CHL protein, which was more soluble in the supernatant. The supernatant samples of the six insoluble enzymes were further validated by Western blot (Fig. 3e). Western blot results showed that all enzymes were present in the supernatant samples. This indicated that pNP868R, VP4, P5, VP3, VCE, and FCE were soluble but low solubility.

Combining the results of SDS-PAGE and Western blot, the following two conclusions could be drawn. First, the mRNA capping enzymes could be successfully expressed in *E. coli* BL21 (DE3), and *E. coli* BL21 (DE3) could be used as an expression host for mRNA capping enzymes. Second, except for CHL, the solubility of the mRNA capping enzyme was low. Based on the above results, the solubility of the mRNA capping enzyme needed to be further improved.

### 3.4 Improving the solubility with lower induction temperature

**Fig. 4:**
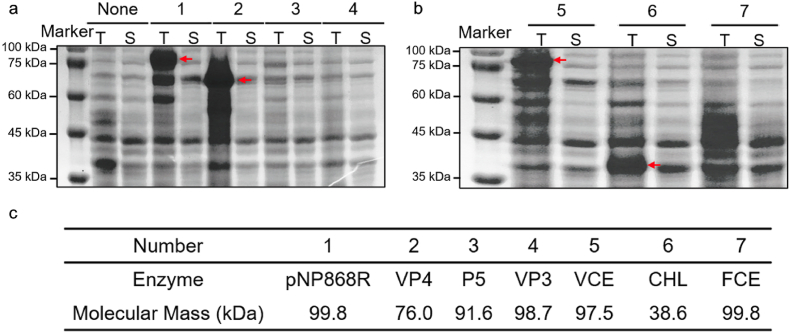
**Expression of mRNA capping enzymes with lower induction temperature**. (a) SDS-PAGE results of mRNA capping enzymes. The ‘T’ represented the total protein sample. The ‘S’ represented the supernatant sample. (b) Different mRNA capping enzymes and their molecular mass.

The first strategy used to increase the solubility of mRNA capping enzymes was lower induction temperature. Induction at a lower temperature was a commonly used method to increase the solubility of proteins. Compared to the original induction conditions, low-temperature induction required a longer time to produce proteins. The original induction condition was induction at 37 °C for 2 h. Here, the lower temperature induction condition was 20 °C for 6 h. The obtained samples were verified by SDS-PAGE (Fig. 4). The results showed that pNP868R, VP4, VCE, and CHL had obvious target protein bands in the whole protein samples, while P5, VP3, and FCE showed no distinguishable bands in the whole protein samples. Meanwhile, none of the supernatant samples showed obvious target protein bands. The above results indicated that only four enzymes, pNP868R, VP4, VCE, and CHL, achieved expression in *E. coli* BL21 (DE3) under lower induction temperatures.

The strategy of lower induction temperature did not improve the solubility of mRNA capping enzymes. Meanwhile, the lower induction temperature strategy inhibited the expression of mRNA capping enzymes instead. The possible reason for this phenomenon was that the activity of some enzymes in *E. coli* BL21 (DE3) was inhibited at a lower temperature, and the expression of mRNA capping enzymes could not be achieved even if the reaction time was extended. Based on the above results, new strategies to improve the solubility of mRNA capping enzymes needed to be found.

### 3.5 Improving the solubility with soluble tags

The second strategy used to increase the solubility of mRNA capping enzymes was soluble tags. These soluble tags not only improved the solubility of target proteins but could also be used as purification tags. The soluble tags were usually added to the N-terminal end of the target protein and were attached to the target protein via a linker. Four soluble tags were selected to investigate their effects on the mRNA capping enzymes (Table 2). These include small ubiquitin-like modifier (SUMO), maltose-binding protein (MBP), thioredoxin A (TrxA), and glutathione-S-transferase (GST).

**Table 2:** Information about the selection of soluble tags.

Soluble tag	Abbreviation	Size	Source
Small ubiquitin-like modifier	SUMO	10.6 kDa	*Escherichia coli*
Maltose-binding protein	MBP	40.3 kDa	*Homo sapiens*
Thioredoxin A	TrxA	11.8 kDa	*Escherichia coli*
Glutathione-S-transferase	GST	25.5 kDa	*Schistosoma japonicum*

SUMO is derived from *E. coli* and has a molecular weight of 10.6 kDa. The most important feature of SUMO tags is that they can be specifically recognized and efficiently degraded by SUMO proteases, making the subsequent tag removal process accurate and efficient. MBP is derived from *Homo sapiens* and has a molecular weight of 40.3 kDa. The most outstanding feature of the MBP label is its wide range of solubilization and high solubilization efficiency. TrxA is derived from *E. coli* and has a molecular weight of 11.8 kDa. The most unique advantage of TrxA labels is their thermal stability. GST is derived from *Schistosoma japonicum* and has a molecular weight of 25.5 kDa. The most outstanding advantage of GST is its ability to develop an affinity with fixed glutathione. The sequences of four solubilizing tags were codon-optimized based on *E. coli* hosts. The lyotropic protein is attached to the N-terminal of the target protein for subsequent experiments.

**Fig. 5:**
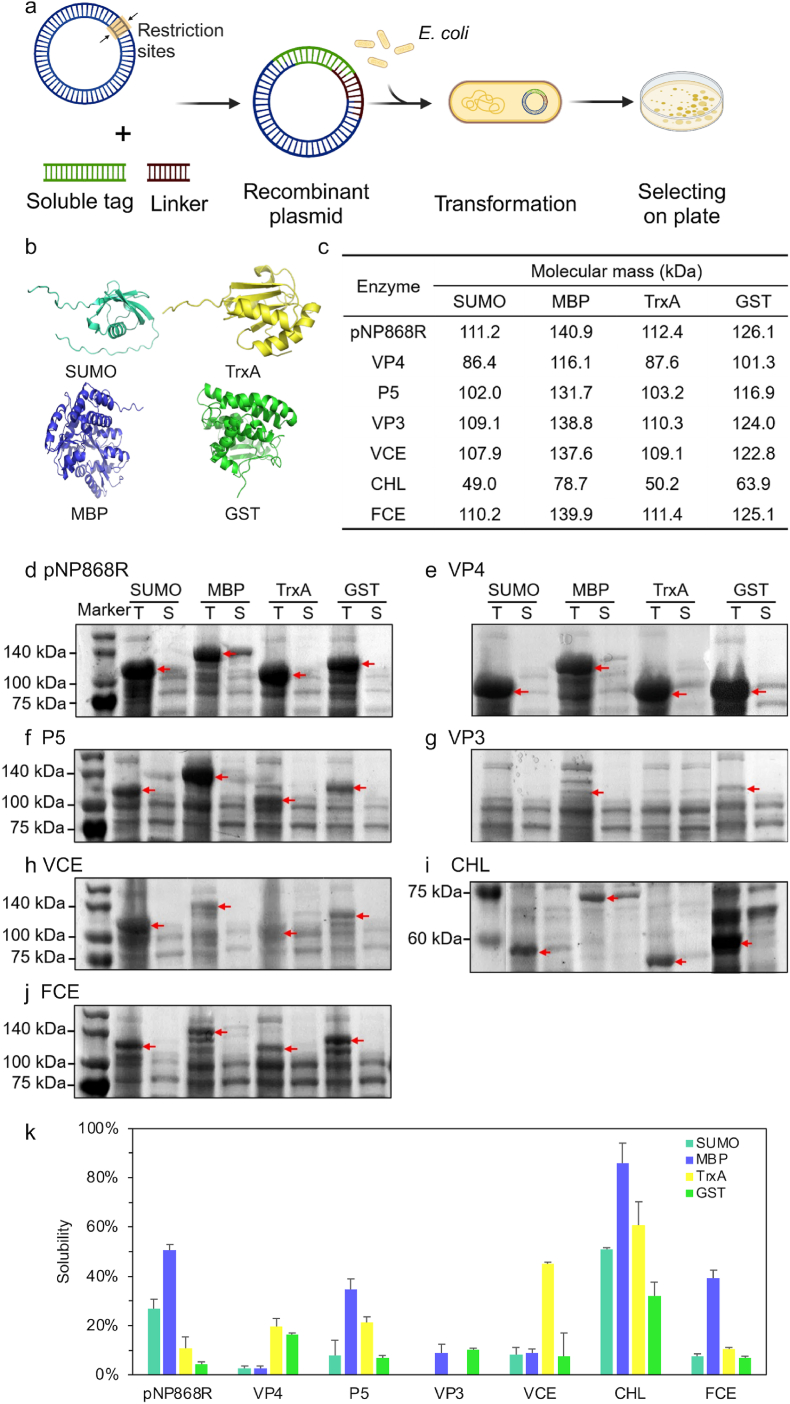
**Expression of mRNA capping enzymes with soluble tags**. The ‘T’ represented the total protein sample. The ‘S’ represented the supernatant sample. (a) Schematic representation of mRNA capping enzyme expression with soluble tags. (b) The structure of four kinds of soluble tags. Predicted by AlphaFold. (c) Different mRNA capping enzymes and their molecular mass. (d) SDS-PAGE results of mRNA capping enzyme pNP868R. (e) SDS-PAGE results of mRNA capping enzyme VP4. (f) SDS-PAGE results of mRNA capping enzyme P5. (g) SDS-PAGE results of mRNA capping enzyme VP3. (h) SDS-PAGE results of mRNA capping enzyme VCE. (i) SDS-PAGE results of mRNA capping enzyme CHL. (g) SDS-PAGE results of mRNA capping enzyme FCE. (k) Soluble analysis of mRNA capping enzyme after adding four kinds of soluble tags.

In order to find a more suitable fusion label, the recombinant expression vector was expressed in *E. coli* BL21 (DE3), and the obtained protein was detected and analyzed using SDS-PAGE (Fig. 5). The experimental results showed that most of the mRNA capping enzymes were highly expressed in the whole protein samples after the fusion of the fusion promotion label, and only VP3 was not fully expressed. This indicated that the addition of the fusion promotion label had no effect on the activity of most enzymes.

Then, the changes in the enzyme solubility after adding different labels were analyzed. The SDS-PAGE results showed that MBP had a good solubilization effect on pNP868R, SUMO had a certain solubilization effect, but TrxA and GST had no obvious solubilization effect. TrxA and GST had some solubilization effect on VP4, but the solubilization effect of SUMO and MBP was not obvious. The solubility of CHL was almost completely dissolved in *E. coli* BL21 (DE3) after the incorporation of MBP, but the incorporation of SUMO, TrxA, and GST reduced the solubility of CHL. MBP and TrxA had some solubilization effects on P5. Only MBP had a certain solubilization effect on FCE and VCE. The four labels had no obvious solubilizing effect on VP3. This meant that there was no single label that applied to all recombinant proteins.

Four different soluble tags were tested, and MBP showed better solubility-enhancing outcomes. However, after fusing with MBP, several enzymes still showed low solubility. The reason why certain soluble tags failed is complicated. The primary structure of a protein - its amino acid sequence - drives the folding and bonding, which ultimately determines the protein's unique three-dimensional structure. Therefore, the sequence properties of these tested 7 enzymes were analyzed by ProtParam (https://web.expasy.org/protparam/), as shown in Table S4. The computational analysis results showed that they revealed obvious differences, including amino acid composition, theoretical pI, and hydropathicity. When these different sequences fuse with MBP sequence, because of the intramolecular folding and bonding of the linear amino acid chain, it certainly could induce different three-dimensional shapes showing different expression and solubility.

Combining the results of the above seven mRNA capping enzymes, the following conclusions could be drawn. First, the soluble effect of the soluble tag on different proteins was quite different, which might inhibit the solubility of the target protein in some cases. Second, among the four selected soluble tags, MBP had the best soluble effect on mRNA capping enzymes. Therefore, the enzymes pNP868P, VP4, CHL, and FCE, which were fused with the soluble tag MBP, were selected for further tests.

### 3.6 Expression of mRNAs in mammalian cells

**Fig. 6:**
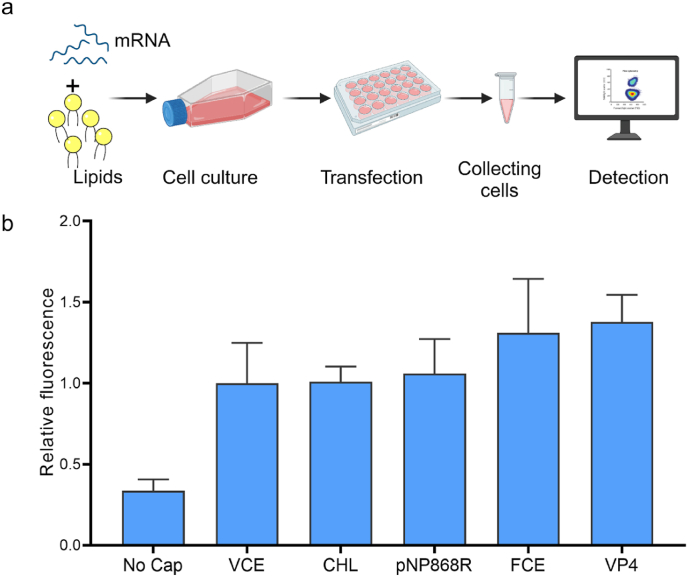
**Expression of mRNAs capped by mRNA capping enzymes in HEK293T**. (a) Schematic diagram of mRNA capping enzyme expression in HEK293T. (b) The relative fluorescence of different mRNAs in HEK293T after being capped by different mRNA capping enzymes. The relative fluorescence selected the result of mRNA capping enzyme VCE as ‘1’.

Because HEK293T is easy to culture and has high transfection efficiency, it is usually used to translate mRNA obtained by transcription *in vitro*. Superfolder green fluorescent protein (sfGFP) was chosen as the target protein for mRNA translation, which made it relatively easy to observe the expression level. After transfected into cells (Fig. 6), it could be found that, compared to the control without enzyme treatment, the mRNA expression levels of VCE, pNP868R, VP4, CHL, and FCE were significantly increased.

In addition, comparing the already widely used VCE with other enzymes, it could be found that the mRNA expression levels by enzymes VP4 and FCE were about 1.3 times higher than that of VCE. The expression level of pNP868R was the same as that of VCE, while the CHL was slightly lower than that of VCE. These results indicated that VP4 and FCE had higher efficiency than VCE and were good enzyme candidates for future studies.

## 4 Conclusion

Cap structure modification is a common method to improve mRNA expression efficiency, which can be achieved by mRNA capping enzyme. Screening mRNA capping enzymes from different virus sources could make mRNA more efficient and improve its translation efficiency. In this study, *E*. *coli* BL21 (DE3) was used to express the enzymes, and to improve the enzyme solubility, low-temperature induction and fusion-promoting labeling strategies were selected. It was found that soluble label MBP had the best promotion effect and could improve the solubility of most enzymes. HEK293T-based mammalian cell protein expression system was used for mRNA translation expression. The results showed that the enzymes derived from bluetongue virus and faustovirus had better activity.

Therefore, this study is significant for more efficient and economical production of mRNA with high expression. However, the process is complicated, which easily leads to mRNA loss and degradation, and the capping efficiency of mRNA with different lengths and compositions is different. mRNA with translation ability is usually longer than other types of RNAs. Therefore, mRNA has a complicated secondary structure, which cannot be ignorable. This structure affects the interaction of mRNA with capping enzymes, further affecting the enzyme efficiency. The secondary structure is closely related to the sequence composition and length. Therefore, it is necessary to further explore how genes and transcription factors participate in the regulation of capping, simplify the whole process, and improve the efficiency of different types and lengths of mRNA. In addition, frontier strategies can be developed to control the entire process, improving its efficiency and enhancing its specificity.

Another challenge for mRNA study is mRNA capping enzyme activity analysis. Activity and substrate specificity analysis is critical for enzymes. However, current technology approaches limit this analysis. The key issue is the problem of RNA being easily degraded. Once RNA samples are injected or input into the instruments, RNAs are immediately degraded, and we cannot get trustable results. That is why most mRNA catalytic studies do not show enzymatic dynamic or interaction data. In short, the limitations of current technological means make it difficult to conduct research on enzyme activity and other aspects. Therefore, in the future, this is a challenging problem that continues to be solved in this field in the future.

## CRediT authorship contribution statement

**Yiming Wang:** Writing – review & editing, Writing – original draft, Methodology, Investigation, Formal analysis, Data curation. **Xiaoxue Wang:** Methodology, Investigation, Data curation. **Wenchao Li:** Methodology, Investigation, Formal analysis. **Xinjie Chen:** Writing – original draft, Methodology, Investigation. **Yuan Lu:** Writing – review & editing, Supervision, Funding acquisition, Conceptualization.

## Data availability statement

All data and materials are available in the manuscript and supporting information.

## Declaration of competing interest

The authors declare that they have no known competing financial interests or personal relationships that could have appeared to influence the work reported in this paper. The author Yuan Lu is an Editor-in-Chief for Biotechnology Notes and was not involved in the editorial review or the decision to publish this article.

## Abbreviations:

3′-UTR: 3′-untranslated regions; 5′-UTR: 5′-untranslated regions; *E. coli: Escherichia coli*; GST: Glutathione-S-transferase; N7 MTase: Guanine-N7 methyltransferase; GTase: Guanylyltransferase; MBP: maltose-binding protein; mRNA: messenger RNA; ORF: open reading frame; *P. pastoris*: *Pichia pastoris*; SUMO: small ubiquitin-like modifier; sfGFP: superfolder green fluorescent protein; TrxA: thioredoxin A; TPase: Triphosphatase; VCE: vaccinia virus capping enzyme.
