# Supplementary material for: A comparative exploration of mRNA capping enzymes
Source: Biotechnol Notes. 2024 Nov 20;5:165–72. doi: 10.1016/j.biotno.2024.11.005 (PMC11625350; doi:10.1016/j.biotno.2024.11.005)
Supplement: Multimedia component 1 [file mmc1.docx]

Supporting Information

**A comparative exploration of mRNA capping enzymes**

*Yiming Wang^1,2^, Xiaoxue Wang^1,2^, Wenchao Li^1,2^, Xinjie Chen^1,2^, Yuan Lu^1,2,*^*

^1^Department of Chemical Engineering, Tsinghua University, Beijing 100084, China.

^2^Key Laboratory of Industrial Biocatalysis, Ministry of Education, Tsinghua University, Beijing 100084, China.

* **Correspondence:** yuanlu@tsinghua.edu.cn (Yuan Lu)

# Methods

***E. coli* protein expression and purification**

The protein expression operations based on *E. coli* are as follows. Target *E. coli* was inoculated into LB liquid medium containing resistance and cultured overnight at 37 ℃ and 220 rpm to obtain primary seed liquid. Then, the primary seed solution was inoculated into LB liquid medium containing resistance at 2%~5% inoculating amount, and cultured at 37 ℃ and 220 rpm to OD_600_ of about 0.6~0.8. Added 0.1% of the inducer. In this study, it was isopropyl-B-D-thiogalactoximab (IPTG) with a final concentration of 1 mM. Cultured at 37 ℃ 220 rpm for 4 h or overnight at 30 ℃ 220 rpm. Centrifuged at 8000 × g for 12 min. Discarded the supernatant and collected the bacterial solution. The bacteria were washed with PBS for 3 times, and the centrifugation condition was 8000 × g for 12 min. Until the supernatant was clear and colorless after centrifugation. Frozen in a -80 ℃ refrigerator or directly for protein purification.

In this study, His GraviTrap (GE Healthcare) was used for protein purification. Prepared the buffers required for protein purification: Binding buffers and Elution buffers. The Binding Buffer consisted of 3.2788 g Na_3_PO_4_, 29.22 g NaCl, 1.362 g Imidazole, and pH 7.4. The Elution Buffer consisted of 3.2788 g Na_3_PO_4_, 29.22 g NaCl, 34.05 g Imidazole, and pH 7.4. All of the above solutions needed to be pumped and filtered with a water filter membrane with a 0.45μm aperture before use.

In this study, all steps of protein purification were required to be performed under low-temperature conditions, and the specific operations were as follows. The bacterial mud was re-suspended with Binding Buffer, so that the final OD_600_ was 40-60. The cells were broken by ultra-low temperature and high-pressure cell crusher (pressure 1200 bar) or ultrasonic cell crusher (power 35%, working interval of 6 s for 2s, total time 40 min). The crushed mixture was centrifuged for 10 min at a low temperature of 12,000 rpm. The supernatant was extracted by an aqueous filter membrane with a pore size of 0.45μm. Balanced His GraviTrap in advance with a 10 mL Binding Buffer. The extracted supernatant was injected into His GraviTrap. After the solution had completely flowed through His GraviTrap, 10 mL Binding Buffer was added to His GraviTrap to wash away the excess impurities. The adsorption column was eluted with 3 mL Elution Buffer, and the collected solution was obtained. His GraviTrap was regenerated according to the instructions for easy reuse. The corresponding protein Storage Buffer was used to dialysis the protein. The protein solution after dialysis was collected and stored in the -20 ℃ or -80 ℃ refrigerator for later use.

**Protein expression based on *Pichia pastoris***

The protein expression operation based on *P. pastoris* was as follows. The target *P. pastoris* strain was selected and inoculated in 10 mL BMGY medium (50 mL shaker). Cultured at 30 ℃ and 250 rpm until OD_600_ was 2~6, about 16~18 h. BMGY bacteria solution was added into 25 mL BMMY medium (250 mL shaker) so that the initial OD_600_ was about 1. If the concentration of BMGY bacterial solution was low, it was necessary to centrifuge the BMGY bacterial solution at 3000 × g for 3 min. The supernatant was discarded, and the bacteria were re-suspended with 1 mL BMMY medium and then added to the BMMY medium. Cultured at 30 ℃, 250 rpm. Added 1% (v/v) methanol every 24 h, or 200 μL if necessary to measure OD_600_. After fermentation for 72 h, the bacteria were collected for the follow-up experiment.

**SDS-PAGE**

The specific operation of sodium dodecyl sulfate-polyacrylamide gel electrophoresis was as follows. The protein sample was evenly mixed with 6×Protein Loading Buffer at the ratio of 5:1. Incubated at 99 ℃ for 10 min. Filled the bath with an electrophoresis solution about half the height of the bath (formula: 25 mM Tris, 250 mM Glycine, 1% SDS), fixed the glue plate, and inserted it into the bath. Added the electrophoresis solution to the inside of the rubber plate until the electrophoresis solution overflowed from the top of the rubber plate, and ensured that the electrophoresis solution inside the rubber plate was higher than the electrophoresis solution outside the rubber plate.

Then, the treated protein sample was added into the glue hole with a pipette gun, and the amount of addition was determined by the size of the glue hole. At the same time, it was also necessary to add a marker into a separate colloidal hole for subsequent determination of protein molecular weight. Powered on, and set voltage 90 V, current 200 mA, and time 30 min. After the sample strip left the concentrated glue, set the voltage to 120 V, the current to 200 mA, and the time to 1 h. When the sample strip reached the bottom of the rubber plate, turned off the power supply. Removed the plate and transferred the gel to a glass culture dish or glass beaker. Added deionized water until it reached the surface of the gel. Microwaved to boil for 30 s. Shaked at 40~60 rpm for 10 min. Disposed of deionized water. Added Coomassie brilliant Blue dye until it was below the surface of the polyacrylamide gel. Microwaveed to boil for 30 s. Decolorization was performed at 40 to 60 rpm for 10 min. Recycleed the Coomassie brilliant blue dye. Added ionized water until it reached the surface of the gel. Microwaved to boil for 30 s. Shaked overnight at 40 to 60 rpm. The next day, gel imaging was performed to check the results.

**Western blotting**

The relevant solution components required for protein immunoblotting were shown in **Table S1**.

The milk-sealing liquid should be stirred for more than 1 h before use. The primary and secondary buffers needed to be stored in a -20 °C refrigerator and could be reused up to 4 times.

Protein immunoblotting required the sample to be subjected to the gel electrophoresis first, but did not stain. The specific steps are as follows.

First, removed the polyacrylamide gel after the power was turned off and cleaned it with deionized water. Prepared the membrane clamp and related equipment in advance, and soaked in the membrane solution. The stacking sequence from bottom to top was film clip, sponge pad, and absorbent filter paper. Made sure the applicator was fully soaked with the applicator solution. Laid the polyacrylamide gel on the top of the absorbent filter paper, and noted the direction of the gel for subsequent operation. PVDF membrane was activated with methanol and laid on polyacrylamide gel. Tried to ensure that the size of the PVDF membrane was consistent with the size of the polyacrylamide gel. If there were air bubbles between the PVDF membrane and the polyacrylamide gel, brushed carefully to empty the air bubbles. After that, the PVDF membrane was successively covered with absorbent filter paper and a sponge pad, and the rotating film was clamped on.

Then, poured the transfer liquid into the transfer tank. Put in the clip and noted the direction of the clip. At the same time, put into the ice tank to avoid excessive temperature during the transfer process. Placed the transfer tank in an ice bath. Powered on, and set voltage 120 V, current 300 mA, and time 2 h.

After the transfer, turned off the power. Removed the PVDF membrane and disposed of the polyacrylamide gel. All subsequent operations required that the protein-containing side of the PVDF membrane faced up, *i.e.* the side in contact with the polyacrylamide gel. PVDF membrane was placed in methanol, activated for 30 seconds, and then stained in the buffer until stained red protein bands appeared on the PVDF membrane. Finally, the PVDF membrane was cleaned with deionized water. If necessary, the cleaned PVDF film could be placed in a plastic bag and the target strip could be cut. The PVDF membrane was immersed in milk sealer. Incubated in a shaker for 1 h at room temperature.

After closure, PVDF membrane was immersed in a buffer solution. Incubated at room temperature for 2 h or in refrigerator at 4 ℃ overnight. After incubation, PVDF membrane was soaked in TBST buffer and washed three times for 10 min each time.

The washed PVDF membrane was immersed in the secondary buffer. Incubated on a shaker for 30 min at room temperature. After incubation, PVDF membrane was soaked in TBST buffer and washed three times for 10 min each time. After washing, the PVDF film was placed in the imager, the exposure liquid was added for exposure, and the experimental results were checked.

# Tables

## Table S1. Western blotting-related solution components.

| Solutions | Components |
| --- | --- |
| Electrophoretic solution | 25 mM Tris, 250 mM Glycine, 0.1% SDS |
| Transfer fluid | 25 mM Tris, 0.2 M Glycine, 20% (v/v) Methanol |
| TBST buffer | 20 mM Tris, 0.2M Nacl, 0.1% (v/v) Tween-20, pH 7.6 |
| Ponceau buffer | 0.1% (w/v) Ponceau, dissolves in 5% (v/v) Acetic Acid |
| Milk sealer | 5% (w/v) milk powder, dissolves in TBST buffer |
| Primary antibody buffer | 0.1% Anti-his Tag Monoclonal antibody, dissolves in milk sealer |
| Secondary antibody buffer | 0.02% HRP AffiniPure Goat Anti-Mouse IgG (H+L), dissolves in TBST buffer |

## Table S2. Amino acid sequences of mRNA capping enzymes from different viral sources.

| Name | Amino acid sequence | |
| --- | --- | --- |
| pNP868R | | MASLDNLVARYQRCFNDQSLKNSTIELEIRFQQINFLLFKTVYEALVAQEIPSTISHSIRCIKKVHHENHCREKILPSENLYFKKQPLMFFKFSEPASLGCKVSLAIEQPIRKFILDSSVLVRLKNRTTFRVSELWKIELTIVKQLMGSEVSAKLAAFKTLLFDTPEQQTTKNMMTLINPDDEYLYEIEIEYTGKPESLTAADVIKIKNTVLTLISPNHLMLTAYHQAIEFIASHILSSEILLARIKSGKWGLKRLLPQVKSMTKADYMKFYPPVGYYVTDKADGIRGIAVIQDTQIYVVADQLYSLGTTGIEPLKPTILDGEFMPKKKEFYGFDVIMYEGNLLTQQGFETRIESLSKGIKVLQAFNIKAEMKPFISLTSADPNVLLKNFESIFKKKTRPYSIDGIILVEPGNSYLNTNTFKWKPTWDNTLDFLVRKCPESLNVPEYAPKKGFSLHLLFVGISGELFKKLALNWCPGYTKLFPVTQRNQNYFPVQFQPSDFPLAFLYYHPDTSSFSNIDGKVLEMRCLKREINYVRWEIVKIREDRQQDLKTGGYFGNDFKTAELTWLNYMDPFSFEELAKGPSGMYFAGAKTGIYRAQTALISFIKQEIIQKISHQSWVIDLGIGKGQDLGRYLDAGVRHLVGIDKDQTALAELVYRKFSHATTRQHKHATNIYVLHQDLAEPAKEISEKVHQIYGFPKEGASSIVSNLFIHYLMKNTQQVENLAVLCHKLLQPGGMVWFTTMLGEQVLELLHENRIELNEVWEARENEVVKFAIKRLFKEDILQETGQEIGVLLPFSNGDFYNEYLVNTAFLIKIFKHHGFSLVQKQSFKDWIPEFQNFSKSLYKILTEADKTWTSLFGFICLRKNHHHHHHHH |
| VP4 | | MPEPHAVLYVTNELSHLVKSGYLPIWHLTGDESLNDLWLENGKYATDVYAYGDVSKWTIRQLRGHGFIFISTHKSVQLADIIKTVDVRVSREVVKSQDMKMLENEIGRRRIRMRKGFGDALRSYAFKVAIEFHGSEAETLNDANPRLHKVYGMPETPPLYMEYAEIGNKFDDEPTDEKLVSMLDYIIYSAEEIHYVGCGDLRTLMQFKKRSPGRFKRVLWHVYDPIAPQCPDTNVIVHNVMVDSKKDILKHINFLKRVERLFIWDVSSDRNQMDDDEWESTRFAEDRLGEEIAYEM  GGAFSSALIKHRVPAKRDEYHCISTYLLPQPGADKDMYELRNFMKLKGYSHVDRHMHPDAAVMKVVSRDVRRMVEMFHGKDRGRFLKKRIFEHLHIIRKNGLFHESDEPRADLFYLTNRCNMGLEPSIYEVMKKSTIATVWVGRTPLYDYDDYSLPRSTVMLNGSYRDIRVLDGNGAILFLMWKYPDIIKKDLTYDPAWAMNFAVSLKEPIPDPPVPDISLCRFIGLRVESSVLRVRNPTLHETADELKRMGLDLSGHLYVTLMSGAYVTDLFWWFKMILEWSSQGKEQKIRDLKRSAAEVIEWKEQMAERPWHVRNDLIAALREYKRKMGTREGASIDSWLELLRHLHHHHHHHH |
| P5 | | MGGSMSNPDYCIPNFSQTVNERTIIDIFTICRYRSPLVVFCLSHNELAKKYAQDVSMSSGTHVHIIDGSVEITVSLYRTFRTIATQLLGRMQIVVFVTVDKSVVSTQVMKSIAWAFRGSFVELRNQSVDSSTLVSKLENLVSFAPLYNVPKCGPDYYGPTVYSELLSLATNARTHWYATIDYSMFTRSVLTGFVAKYFNEEAVPIDKRIVSIVGYNPPYVWTCLRHGIRPTYIEKSLPNPGGKGPFGLILPVINELVLKSKVKYVMHNPQIKLLCLDTFMLSTSMNILYIGAYPATHLLSLQLNGWTILAFDPKITSDWTDAMAKATGAKVIGVSKEFDFKSFSVQANQLNMFQNSKLSVIDDTWVETDYEKFQSEKQAYFEWLIDRTSIDVRLISMKWNRSKDTSVSHLLALLPQPYGASIREMRAFFHKKGASDIKILAAETEKYMDDFTAMSVSDQINTQKFMHCMITTVGDALKMDLDGGRAVIASYSLSNSSNSKERVLKFLSDANKAKAMVVFGAPNTHRLAYAKKVGLVLDSAIKMSKDLITFSNPTGRRWRDYGYSQSELYDAGYVEITIDQMVAYSSDVYNGVGYFANSTYNDLFSWYIPKWYVHKRMLMQDIRLSPAALVKCFTTLIRNICYVPHETYYRFRGILVDKYLRSKNVDPSQYSIVGSGSKTFTVLSHFEVPHECGPLVFEASTDVNISGHLLSLAIAAHFVASPMILWAEQMKYMAVDRMLPPNLDKSLFFDNKVTPSGALQRWHSREEVLLAAEICESYAAMMLNNKHSPDIIGTLKSAINLVFKIHHHHHHHH |
| VP3 | | MKVLALRHSVAQVYADTQVYTHDDSKDEYENAFLISNLTTHNILYLNYNVKTLQILNKSGIAAIEIQKIDELFTLIRCNFTYDYTDDVVYLHDYSYYTNNEIRTDQHWITKTNIEDYLLPGWKLTYVGYNGSDTRGHYNFSFRCQNAATDDDAIIEYIYSDELDFQSFILKKI  KERMTTSLPIARLSNRVFRDKLFKTLSANHDKVVNIGPRNESMFTFLDYPSIRQFSNGPYLVKDTIKLKQERWLGKRLSQFDIGQYKNMLNVLTTLYQYYNIYHEKPIVYMIGSAPSYWIYDIKQYSNLKFETWDPLDTPYSNLHHKELFYINDVKKLKDNSILYIDIRTDRGTVDWKEWRKIVERQTIDNLHIAYKYLSTGKAKVCCVKMTAMDLELPISAKLLHHPTTEIRSEFYLVMDIWDSKNIKRFIPKGVLYSYINNTITENVFIQQPFKLKTLKNECIIALYALSNDLNNREDVVKLINNQKKALMTVRINNTFKDEPKVGFKNIYDWTFLPTDFETNGSIITSYDGCLGIFGLSISLASKPTGNNHLFILSGTDKYFKLDQFANHMSISRRSHQIRFSESATSYSGYIFRDLSNNNFNLIGTNVENSVSGHVYNALIYYRYNYSFDLKRWIYLHSTGKASIEGGKYYEHAPIELIYACRSAREFAKLQDDLTVLRYSNEIENYINKVYSITYADDPNYFIGVKFKNIPYKYNVKVPHLTFGVLNISEQMLPDVITILKRFKNELFGMEITTSYTYMLSDEVYVANISGVLSTYFKIYNAFYKEQITFGQSRMFIPHVTLSFSNEKTVRIDTTKLYIDSIYLRKIKGDTVFDMTGHHHHHHHH |
| VCE | | MDANVVSSSTIATYIDALAKNASELEQRSTAYEINNELELVFIKPPLITLTNVVNISTIQESFIRFTVTNKEGVKIRTKIPLSKVHGLDVKNVQLVDAIDNIVWEKKSLVTENRLHKECLLRLSTEERHIFLDYKKYGSSIRLELVNLIQAKTKNFTIDFKLKYFLGSGAQSKSSLL  HAINHPKSRPNTSLEIEFTPRDNETVPYDELIKELTTLSRHIFMASPENVILSPPINAPIKTFMLPKQDIVGLDLENLYAVTKTDGIPITIRVTSNGLYCYFTHLGYIIRYPVKRIIDSEVVVFGEAVKDKNWTVYLIKLIEPVNAINDRLEESKYVESKLVDICDRIVFKSKKYEGPFTTTSEVVDMLSTYLPKQPEGVILFYSKGPKSNIDFKIKKENTIDQTANVVFRYMSSEPIIFGESSIFVEYKKFSNDKGFPKEYGSGKIVLYNGVNYLNNIYCLEYINTHNEVGIKSVVVPIKFIAEFLVNGEILKPRIDKTMKYINSEDYYGNQHNIIVEHLRDQSIKIGDIFNEDKLSDVGHQYANNDKFRLNPEVSYFTNKRTRGPLGILSNYVKTLLISMYCSKTFLDDSNKRKVLAIDFGNGADLEKYFYGEIALLVATDPDADAIARGNERYNKLNSGIKTKYYKFDYIQETIRSDTFVSSVREVFYFGKFNIIDWQFAIHYSFHPRHYATVMNNLSELTASGGKVLITTMDGDKLSKLTDKKTFIIHKNLPSSENYMSVEKIADDRIVVYNPSTMSTPMTEYIIKKNDIVRVFNEYGFVLVDNVDFATIIERSKKFINGASTMEDRPSTRNFFELNRGAIKCEGLDVEDLLSYYVVYVFSKRHHHHHHHH |
| CHL | | MVPPTINTGKNITTERAVLTLNGLQIKLHKVVGESRDDIVAKMKDLAMDDHKFPRLPGPNPVSIERKDFEKLKQNKYVVSEKTDGIRFMMFFTRVFGFKVCTIIDRAMTVYLLPFKNIPRVLFQGSIFDGELCVDIVEKKFAFVLFDAVVVSGVTVSQMDLASRFFAMKRSLKEFKNVPEDPAILRYKEWIPLEHPTIIKDHLKKANAIYHTDGLIIMSVDEPVIYGRNFNLFKLKPGTHHTIDFIIMSEDGTIGIFDPNLRKNVPVGKLDGYYNKGSIVECGFADGTWKYIQGRSDKNQANDRLTYEKTLLNIEENITIDELLDLFKWEHHHHHHHH |
| FCE | | MAKRLQRCQDVNQVCEIYNSKGGIGELELRFDKLPQNLFAGVFDKLKPDGEIQTTMRVSNRDGVAREITFGGGVKTNEIFVKKQNICVFDVVDIFSYKVAVSTEETVVEKPTMETTAGVRFKIRLSVEDVVKDWRIDLTAVKTAELGKIAQHTASIVQRTFPDNLLKLTGAEVAKLAADSYELELEYTGKSPATNEKVNVAAKYAVELLSSVRNANSTAAASFGESVSDLCRVAKIIHTHEYANVVCRTPSFKMLLPQVVSLTKSSYYGGLYPPENLWLAGKTDGVRALVVCEDGVAKVITAESVDITHGVCSATTILDCELNVDAKILYVFDVIISNNTQVYFFFFFFFFTQPFSTRITTDISDIKIDGYKIEMKPFVKVVKADEATFKSAYKAPHNEGLIMIEDGAAYAATKTYKWKPLSHNTIDFLIKACPKQLINVDPYKPRAGYKLWLLFTTISLDQQRELGIEFIPAWKILFTDINMFGSRVPIQFQPAINPLAYVCYLPEDVNVNDGDIVEMRAVDGYDTIPKWELVRSRNDRKNEPGFYGNNYKIASDIYLNYIDVFHFEDLYKYNPGYFEKNKSDIYVAPNKYRRYLIKSLFGRYLRDAKWVIDAAAGRGADLHLYKAECVEHLLAIDIDPTAISELVRRRNEITGYNKSHRGGRNMHSHRGQSHCAKSTSLHALVADLRENPDVLIPKIIQSRPHERCYDAIVINFAIHYLCDTDEHIRDFLITVSRLLAPNGVFIFTTMDGESIVKLLADHKVRPGEAWTIHTGDVNSPDSTVPKYSIRRLYDSDKLTKTGQQIEVLLPMSGEMKAEPLCNIKNIISMARKMGLDLVESANFSVLYEAYARDYPDIYARMTPDDKLYNDLHTYAVFKRKKGASATSHHHHHHHH |

## Table S3. The nucleic acid sequence and amino acid sequence of the soluble tags.

| Soluble tag | Abbreviation | Nucleic acid sequence | Amino acid sequence |
| --- | --- | --- | --- |
| Small ubiquitin-like modifier | SUMO | atggccgacgaaaagcccaaggaaggagtcaagactgagaacaacgatcatattaatttgaaggtggcggggcaggatggttctgtggtgcagtttaagattaagaggcatacaccacttagtaaactaatgaaagcctattgtgaacgacagggattgtcaatgaggcagatcagattccgatttgacgggcaaccaatcaatgaaacagacacacctgcacagttggaaatggaggatgaagatacaattgatgtgttccaacagcagacgggaggt | MSDQEAKPSTEDLGDKKEGEYIKLKVIGQDSSEIHFKVKMTTHLKKLKESYCQRQGVPMNSLRFLFEGQRIADNHTPKELGMEEEDVIEVYQEQTGGGGGGSGGGGS |
| Maltose-binding protein | MBP | atgaaaatcgaagaaggtaaactggtaatctggattaacggcgataaaggctataacggtctcgctgaagtcggtaagaaattcgagaaagataccggaattaaagtcaccgttgagcatccggataaactggaagagaaattcccacaggttgcggcaactggcgatggccctgacattatcttctgggcacacgaccgctttggtggctacgctcaatctggcctgttggctgaaatcaccccggacaaagcgttccaggacaagctgtatccgtttacctgggatgccgtacgttacaacggcaagctgattgcttacccgatcgctgttgaagcgttatcgctgatttataacaaagatctgctgccgaacccgccaaaaacctgggaagagatcccggcgctggataaagaactgaaagcgaaaggtaagagcgcgctgatgttcaacctgcaagaaccgtacttcacctggccgctgattgctgctgacgggggttatgcgttcaagtatgaaaacggcaagtacgacattaaagacgtgggcgtggataacgctggcgcgaaagcgggtctgaccttcctggttgacctgattaaaaacaaacacatgaatgcagacaccgattactccatcgcagaagctgcctttaataaaggcgaaacagcgatgaccatcaacggcccgtgggcatggtccaacatcgacaccagcaaagtgaattatggtgtaacggtactgccgaccttcaagggtcaaccatccaaaccgttcgttggcgtgctgagcgcaggtattaacgccgccagtccgaacaaagagctggcaaaagagttcctcgaaaactatctgctgactgatgaaggtctggaagcggttaataaagacaaaccgctgggtgccgtagcgctgaagtcttacgaggaagagttggcgaaagatccacgtattgccgccactatggaaaacgcccagaaaggtgaaatcatgccgaacatcccgcagatgtccgctttctggtatgccgtgcgtactgcggtgatcaacgccgccagcggtcgtcagactgtcgatgaagccctgaaagacgcgcagact | MKIEEGKLVIWINGDKGYNGLAEVGKKFEKDTGIKVTVEHPDKLEEKFPQVAATGDGPDIIFWAHDRFGGYAQSGLLAEITPDKAFQDKLYPFTWDAVRYNGKLIAYPIAVEALSLIYNKDLLPNPPKTWEEIPALDKELKAKGKSALMFNLQEPYFTWPLIAADGGYAFKYENGKYDIKDVGVDNAGAKAGLTFLVDLIKNKHMNADTDYSIAEAAFNKGETAMTINGPWAWSNIDTSKVNYGVTVLPTFKGQPSKPFVGVLSAGINAASPNKELAKEFLENYLLTDEGLEAVNKDKPLGAVALKSYEEELAKDPRIAATMENAQKGEIMPNIPQMSAFWYAVRTAVINAASGRQTVDEALKDAQTGGGGSGGGGS |
| Thioredoxin A | TrxA | atgagcgataaaattattcacctgactgacgacagttttgacacggatgtactcaaagcggacggggcgatcctcgtcgatttctgggcagagtggtgcggtccgtgcaaaatgatcgccccgattctggatgaaatcgctgacgaatatcagggcaaactgaccgttgcaaaactgaacatcgatcaaaaccctggcactgcgccgaaatatggcatccgtggtatcccgactctgctgctgttcaaaaacggtgaagtggcggcaaccaaagtgggtgcactgtctaaaggtcagttgaaagagttcctcgacgctaacctggcc | MSDKIIHLTDDSFDTDVLKADGAILVDFWAEWCGPCKMIAPILDEIADEYQGKLTVAKLNIDQNPGTAPKYGIRGIPTLLLFKNGEVAATKVGALSKGQLKEFLDANLAGGGGSGGGGS |
| Glutathione-S-transferase | GST | atgtcccctatactaggttattggaaaattaagggccttgtgcaacccactcgacttcttttggaatatcttgaagaaaaatatgaagagcatttgtatgagcgcgatgaaggtgataaatggcgaaacaaaaagtttgaattgggtttggagtttcccaatcttccttattatattgatggtgatgttaaattaacacagtctatggccatcatacgttatatagctgacaagcacaacatgttgggtggttgtccaaaagagcgtgcagagatttcaatgcttgaaggagcggttttggatattagatacggtgtttcgagaattgcatatagtaaagactttgaaactctcaaagttgattttcttagcaagctacctgaaatgctgaaaatgttcgaagatcgtttatgtcataaaacatatttaaatggtgatcatgtaacccatcctgacttcatgttgtatgacgctcttgatgttgttttatacatggacccaatgtgcctggatgcgttcccaaaattagtttgttttaaaaaacgtattgaagctatcccacaaattgataagtacttgaaatccagcaagtatatagcatggcctttgcagggctggcaagccacgtttggtggtggcgaccatcctccaaaa | MSPILGYWKIKGLVQPTRLLLEYLEEKYEEHLYERDEGDKWRNKKFELGLEFPNLPYYIDGDVKLTQSMAIIRYIADKHNMLGGCPKERAEISMLEGAVLDIRYGVSRIAYSKDFETLKVDFLSKLPEMLKMFEDRLCHKTYLNGDHVTHPDFMLYDALDVVLYMDPMCLDAFPKLVCFKKRIEAIPQIDKYLKSSKYIAWPLQGWQATFGGGDHPPKGGGGSGGGGS |

**Table S4 Protein sequence analysis by ProtParam**

| **Protein** | **Basic information** | **Amino acid composition** | |
| --- | --- | --- | --- |
| pNP868R | Number of amino acids: 868  Molecular weight: 99967.80  Theoretical pI: 8.75  Grand average of hydropathicity: -0.194 | Ala (A) 44 5.1%  Arg (R) 30 3.5%  Asn (N) 39 4.5%  Asp (D) 35 4.0%  Cys (C) 9 1.0%  Gln (Q) 42 4.8%  Glu (E) 60 6.9%  Gly (G) 45 5.2%  His (H) 21 2.4%  Ile (I) 67 7.7% | Leu (L) 96 11.1%  Lys (K) 75 8.6%  Met (M) 17 2.0%  Phe (F) 55 6.3%  Pro (P) 36 4.1%  Ser (S) 53 6.1%  Thr (T) 50 5.8%  Trp (W) 12 1.4%  Tyr (Y) 35 4.0%  Val (V) 47 5.4% |
| VP4 | Number of amino acids: 644  Molecular weight: 75228.51  Theoretical pI: 7.13  Grand average of hydropathicity (GRAVY):  -0.444 | Ala (A) 44 5.1%  Arg (R) 30 3.5%  Asn (N) 39 4.5%  Asp (D) 35 4.0%  Cys (C) 9 1.0%  Gln (Q) 42 4.8%  Glu (E) 60 6.9%  Gly (G) 45 5.2%  His (H) 21 2.4%  Ile (I) 67 7.7% | Leu (L) 96 11.1%  Lys (K) 75 8.6%  Met (M) 17 2.0%  Phe (F) 55 6.3%  Pro (P) 36 4.1%  Ser (S) 53 6.1%  Thr (T) 50 5.8%  Trp (W) 12 1.4%  Tyr (Y) 35 4.0%  Val (V) 47 5.4% |
| P5 | Number of amino acids: 805  Molecular weight: 90831.82  Theoretical pI: 8.77  Grand average of hydropathicity: -0.027 | Ala (A) 55 6.8%  Arg (R) 33 4.1%  Asn (N) 36 4.5%  Asp (D) 43 5.3%  Cys (C) 11 1.4%  Gln (Q) 22 2.7%  Glu (E) 30 3.7%  Gly (G) 38 4.7%  His (H) 19 2.4%  Ile (I) 53 6.6% | Leu (L) 72 8.9%  Lys (K) 50 6.2%  Met (M) 29 3.6%  Phe (F) 38 4.7%  Pro (P) 33 4.1%  Ser (S) 78 9.7%  Thr (T) 48 6.0%  Trp (W) 13 1.6%  Tyr (Y) 40 5.0%  Val (V) 64 8.0% |
| VP3 | Number of amino acids: 835  Molecular weight: 97907.68  Theoretical pI: 8.45  Grand average of hydropathicity: -0.348 | Ala (A) 31 3.7%  Arg (R) 34 4.1%  Asn (N) 62 7.4%  Asp (D) 54 6.5%  Cys (C) 7 0.8%  Gln (Q) 23 2.8%  Glu (E) 38 4.6%  Gly (G) 33 4.0%  His (H) 21 2.5%  Ile (I) 77 9.2% | Leu (L) 75 9.0%  Lys (K) 64 7.7%  Met (M) 15 1.8%  Phe (F) 43 5.1%  Pro (P) 22 2.6%  Ser (S) 58 6.9%  Thr (T) 60 7.2%  Trp (W) 10 1.2%  Tyr (Y) 66 7.9%  Val (V) 42 5.0% |
| VCE | Number of amino acids: 844  Molecular weight: 96733.96  Theoretical pI: 7.88  Grand average of hydropathicity: -0.251 | Ala (A) 32 3.8%  Arg (R) 34 4.0%  Asn (N) 59 7.0%  Asp (D) 49 5.8%  Cys (C) 6 0.7%  Gln (Q) 13 1.5%  Glu (E) 57 6.8%  Gly (G) 38 4.5%  His (H) 15 1.8%  Ile (I) 80 9.5% | Leu (L) 69 8.2%  Lys (K) 74 8.8%  Met (M) 13 1.5%  Phe (F) 44 5.2%  Pro (P) 31 3.7%  Ser (S) 63 7.5%  Thr (T) 53 6.3%  Trp (W) 3 0.4%  Tyr (Y) 46 5.5%  Val (V) 65 7.7% |
| CHL | Number of amino acids: 330  Molecular weight: 37832.02  Theoretical pI: 8.23  Grand average of hydropathicity: -0.178 | Ala (A) 13 3.9%  Arg (R) 15 4.5%  Asn (N) 17 5.2%  Asp (D) 25 7.6%  Cys (C) 3 0.9%  Gln (Q) 6 1.8%  Glu (E) 19 5.8%  Gly (G) 20 6.1%  His (H) 7 2.1%  Ile (I) 29 8.8% | Leu (L) 28 8.5%  Lys (K) 31 9.4%  Met (M) 10 3.0%  Phe (F) 22 6.7%  Pro (P) 16 4.8%  Ser (S) 12 3.6%  Thr (T) 19 5.8%  Trp (W) 3 0.9%  Tyr (Y) 9 2.7%  Val (V) 26 7.9% |
| FCE | Number of amino acids: 887  Molecular weight: 100193.85  Theoretical pI: 7.04  Grand average of hydropathicity: -0.202 | Ala (A) 70 7.9%  Arg (R) 45 5.1%  Asn (N) 42 4.7%  Asp (D) 61 6.9%  Cys (C) 15 1.7%  Gln (Q) 20 2.3%  Glu (E) 50 5.6%  Gly (G) 46 5.2%  His (H) 20 2.3%  Ile (I) 65 7.3% | Leu (L) 71 8.0%  Lys (K) 65 7.3%  Met (M) 15 1.7%  Phe (F) 40 4.5%  Pro (P) 37 4.2%  Ser (S) 50 5.6%  Thr (T) 53 6.0%  Trp (W) 8 0.9%  Tyr (Y) 43 4.8%  Val (V) 71 8.0% |

# Figures


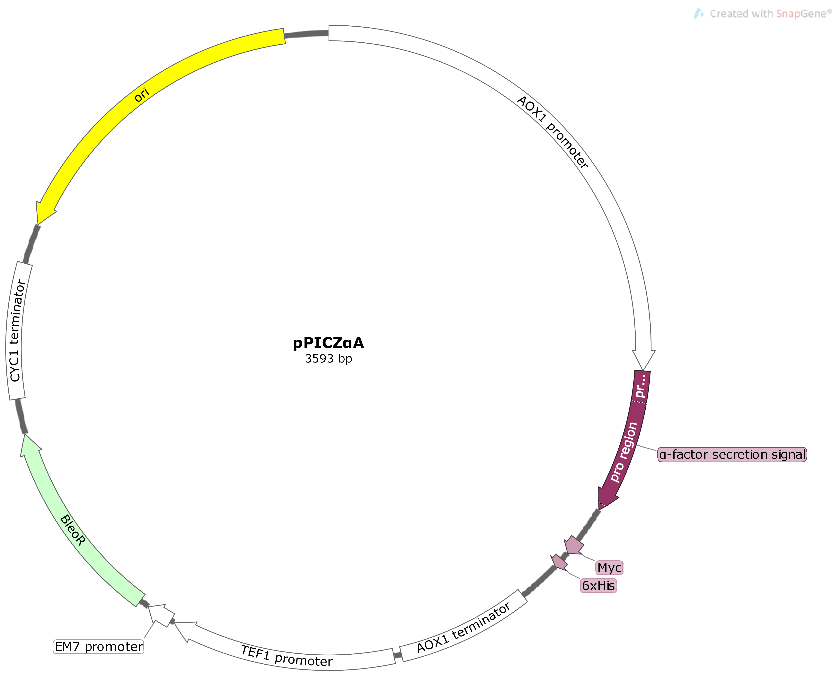


AGATCTAACATCCAAAGACGAAAGGTTGAATGAAACCTTTTTGCCATCCGACATCCACAGGTCCATTCTCACACATAAGTGCCAAACGCAACAGGAGGGGATACACTAGCAGCAGACCGTTGCAAACGCAGGACCTCCACTCCTCTTCTCCTCAACACCCACTTTTGCCATCGAAAAACCAGCCCAGTTATTGGGCTTGATTGGAGCTCGCTCATTCCAATTCCTTCTATTAGGCTACTAACACCATGACTTTATTAGCCTGTCTATCCTGGCCCCCCTGGCGAGGTTCATGTTTGTTTATTTCCGAATGCAACAAGCTCCGCATTACACCCGAACATCACTCCAGATGAGGGCTTTCTGAGTGTGGGGTCAAATAGTTTCATGTTCCCCAAATGGCCCAAAACTGACAGTTTAAACGCTGTCTTGGAACCTAATATGACAAAAGCGTGATCTCATCCAAGATGAACTAAGTTTGGTTCGTTGAAATGCTAACGGCCAGTTGGTCAAAAAGAAACTTCCAAAAGTCGGCATACCGTTTGTCTTGTTTGGTATTGATTGACGAATGCTCAAAAATAATCTCATTAATGCTTAGCGCAGTCTCTCTATCGCTTCTGAACCCCGGTGCACCTGTGCCGAAACGCAAATGGGGAAACACCCGCTTTTTGGATGATTATGCATTGTCTCCACATTGTATGCTTCCAAGATTCTGGTGGGAATACTGCTGATAGCCTAACGTTCATGATCAAAATTTAACTGTTCTAACCCCTACTTGACAGCAATATATAAACAGAAGGAAGCTGCCCTGTCTTAAACCTTTTTTTTTATCATCATTATTAGCTTACTTTCATAATTGCGACTGGTTCCAATTGACAAGCTTTTGATTTTAACGACTTTTAACGACAACTTGAGAAGATCAAAAAACAACTAATTATTCGAAACGATGAGATTTCCTTCAATTTTTACTGCTGTTTTATTCGCAGCATCCTCCGCATTAGCTGCTCCAGTCAACACTACAACAGAAGATGAAACGGCACAAATTCCGGCTGAAGCTGTCATCGGTTACTCAGATTTAGAAGGGGATTTCGATGTTGCTGTTTTGCCATTTTCCAACAGCACAAATAACGGGTTATTGTTTATAAATACTACTATTGCCAGCATTGCTGCTAAAGAAGAAGGGGTATCTCTCGAGAAAAGAGAGGCTGAAGCTGAATTCACGTGGCCCAGCCGGCCGTCTCGGATCGGTACCTCGAGCCGCGGCGGCCGCCAGCTTTCTAGAACAAAAACTCATCTCAGAAGAGGATCTGAATAGCGCCGTCGACCATCATCATCATCATCATTGAGTTTGTAGCCTTAGACATGACTGTTCCTCAGTTCAAGTTGGGCACTTACGAGAAGACCGGTCTTGCTAGATTCTAATCAAGAGGATGTCAGAATGCCATTTGCCTGAGAGATGCAGGCTTCATTTTTGATACTTTTTTATTTGTAACCTATATAGTATAGGATTTTTTTTGTCATTTTGTTTCTTCTCGTACGAGCTTGCTCCTGATCAGCCTATCTCGCAGCTGATGAATATCTTGTGGTAGGGGTTTGGGAAAATCATTCGAGTTTGATGTTTTTCTTGGTATTTCCCACTCCTCTTCAGAGTACAGAAGATTAAGTGAGACCTTCGTTTGTGCGGATCCCCCACACACCATAGCTTCAAAATGTTTCTACTCCTTTTTTACTCTTCCAGATTTTCTCGGACTCCGCGCATCGCCGTACCACTTCAAAACACCCAAGCACAGCATACTAAATTTTCCCTCTTTCTTCCTCTAGGGTGTCGTTAATTACCCGTACTAAAGGTTTGGAAAAGAAAAAAGAGACCGCCTCGTTTCTTTTTCTTCGTCGAAAAAGGCAATAAAAATTTTTATCACGTTTCTTTTTCTTGAAATTTTTTTTTTTAGTTTTTTTCTCTTTCAGTGACCTCCATTGATATTTAAGTTAATAAACGGTCTTCAATTTCTCAAGTTTCAGTTTCATTTTTCTTGTTCTATTACAACTTTTTTTACTTCTTGTTCATTAGAAAGAAAGCATAGCAATCTAATCTAAGGGGCGGTGTTGACAATTAATCATCGGCATAGTATATCGGCATAGTATAATACGACAAGGTGAGGAACTAAACCATGGCCAAGTTGACCAGTGCCGTTCCGGTGCTCACCGCGCGCGACGTCGCCGGAGCGGTCGAGTTCTGGACCGACCGGCTCGGGTTCTCCCGGGACTTCGTGGAGGACGACTTCGCCGGTGTGGTCCGGGACGACGTGACCCTGTTCATCAGCGCGGTCCAGGACCAGGTGGTGCCGGACAACACCCTGGCCTGGGTGTGGGTGCGCGGCCTGGACGAGCTGTACGCCGAGTGGTCGGAGGTCGTGTCCACGAACTTCCGGGACGCCTCCGGGCCGGCCATGACCGAGATCGGCGAGCAGCCGTGGGGGCGGGAGTTCGCCCTGCGCGACCCGGCCGGCAACTGCGTGCACTTCGTGGCCGAGGAGCAGGACTGACACGTCCGACGGCGGCCCACGGGTCCCAGGCCTCGGAGATCCGTCCCCCTTTTCCTTTGTCGATATCATGTAATTAGTTATGTCACGCTTACATTCACGCCCTCCCCCCACATCCGCTCTAACCGAAAAGGAAGGAGTTAGACAACCTGAAGTCTAGGTCCCTATTTATTTTTTTATAGTTATGTTAGTATTAAGAACGTTATTTATATTTCAAATTTTTCTTTTTTTTCTGTACAGACGCGTGTACGCATGTAACATTATACTGAAAACCTTGCTTGAGAAGGTTTTGGGACGCTCGAAGGCTTTAATTTGCAAGCTGGAGACCAACATGTGAGCAAAAGGCCAGCAAAAGGCCAGGAACCGTAAAAAGGCCGCGTTGCTGGCGTTTTTCCATAGGCTCCGCCCCCCTGACGAGCATCACAAAAATCGACGCTCAAGTCAGAGGTGGCGAAACCCGACAGGACTATAAAGATACCAGGCGTTTCCCCCTGGAAGCTCCCTCGTGCGCTCTCCTGTTCCGACCCTGCCGCTTACCGGATACCTGTCCGCCTTTCTCCCTTCGGGAAGCGTGGCGCTTTCTCAATGCTCACGCTGTAGGTATCTCAGTTCGGTGTAGGTCGTTCGCTCCAAGCTGGGCTGTGTGCACGAACCCCCCGTTCAGCCCGACCGCTGCGCCTTATCCGGTAACTATCGTCTTGAGTCCAACCCGGTAAGACACGACTTATCGCCACTGGCAGCAGCCACTGGTAACAGGATTAGCAGAGCGAGGTATGTAGGCGGTGCTACAGAGTTCTTGAAGTGGTGGCCTAACTACGGCTACACTAGAAGGACAGTATTTGGTATCTGCGCTCTGCTGAAGCCAGTTACCTTCGGAAAAAGAGTTGGTAGCTCTTGATCCGGCAAACAAACCACCGCTGGTAGCGGTGGTTTTTTTGTTTGCAAGCAGCAGATTACGCGCAGAAAAAAAGGATCTCAAGAAGATCCTTTGATCTTTTCTACGGGGTCTGACGCTCAGTGGAACGAAAACTCACGTTAAGGGATTTTGGTCATGAGATC

**Figure S1. pPICZalpha A plasmid map.**


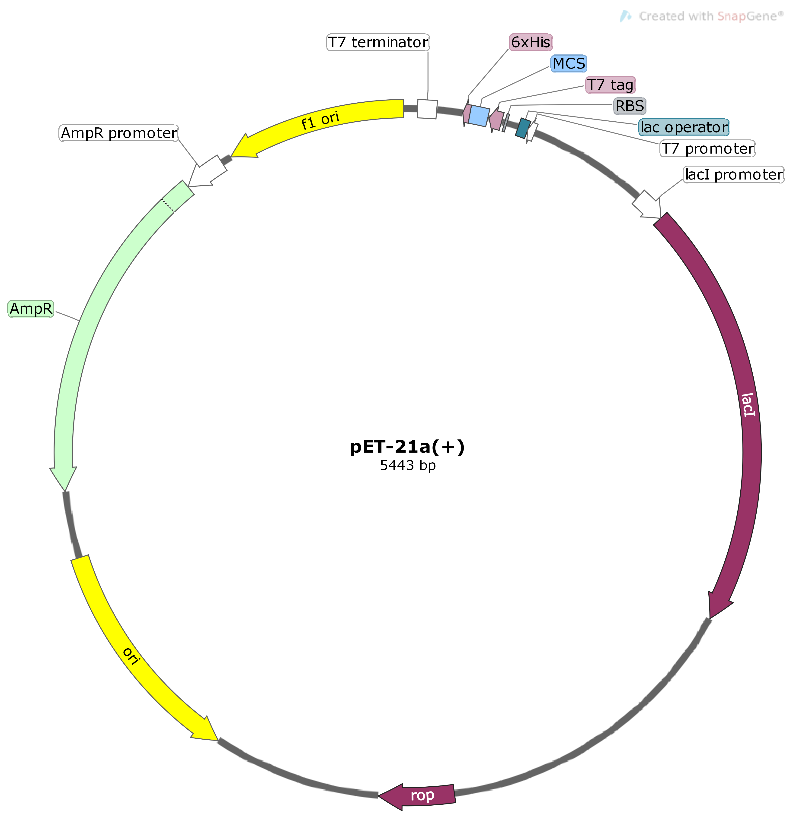


ATCCGGATATAGTTCCTCCTTTCAGCAAAAAACCCCTCAAGACCCGTTTAGAGGCCCCAAGGGGTTATGCTAGTTATTGCTCAGCGGTGGCAGCAGCCAACTCAGCTTCCTTTCGGGCTTTGTTAGCAGCCGGATCTCAGTGGTGGTGGTGGTGGTGCTCGAGTGCGGCCGCAAGCTTGTCGACGGAGCTCGAATTCGGATCCGCGACCCATTTGCTGTCCACCAGTCATGCTAGCCATATGTATATCTCCTTCTTAAAGTTAAACAAAATTATTTCTAGAGGGGAATTGTTATCCGCTCACAATTCCCCTATAGTGAGTCGTATTAATTTCGCGGGATCGAGATCTCGATCCTCTACGCCGGACGCATCGTGGCCGGCATCACCGGCGCCACAGGTGCGGTTGCTGGCGCCTATATCGCCGACATCACCGATGGGGAAGATCGGGCTCGCCACTTCGGGCTCATGAGCGCTTGTTTCGGCGTGGGTATGGTGGCAGGCCCCGTGGCCGGGGGACTGTTGGGCGCCATCTCCTTGCATGCACCATTCCTTGCGGCGGCGGTGCTCAACGGCCTCAACCTACTACTGGGCTGCTTCCTAATGCAGGAGTCGCATAAGGGAGAGCGTCGAGATCCCGGACACCATCGAATGGCGCAAAACCTTTCGCGGTATGGCATGATAGCGCCCGGAAGAGAGTCAATTCAGGGTGGTGAATGTGAAACCAGTAACGTTATACGATGTCGCAGAGTATGCCGGTGTCTCTTATCAGACCGTTTCCCGCGTGGTGAACCAGGCCAGCCACGTTTCTGCGAAAACGCGGGAAAAAGTGGAAGCGGCGATGGCGGAGCTGAATTACATTCCCAACCGCGTGGCACAACAACTGGCGGGCAAACAGTCGTTGCTGATTGGCGTTGCCACCTCCAGTCTGGCCCTGCACGCGCCGTCGCAAATTGTCGCGGCGATTAAATCTCGCGCCGATCAACTGGGTGCCAGCGTGGTGGTGTCGATGGTAGAACGAAGCGGCGTCGAAGCCTGTAAAGCGGCGGTGCACAATCTTCTCGCGCAACGCGTCAGTGGGCTGATCATTAACTATCCGCTGGATGACCAGGATGCCATTGCTGTGGAAGCTGCCTGCACTAATGTTCCGGCGTTATTTCTTGATGTCTCTGACCAGACACCCATCAACAGTATTATTTTCTCCCATGAAGACGGTACGCGACTGGGCGTGGAGCATCTGGTCGCATTGGGTCACCAGCAAATCGCGCTGTTAGCGGGCCCATTAAGTTCTGTCTCGGCGCGTCTGCGTCTGGCTGGCTGGCATAAATATCTCACTCGCAATCAAATTCAGCCGATAGCGGAACGGGAAGGCGACTGGAGTGCCATGTCCGGTTTTCAACAAACCATGCAAATGCTGAATGAGGGCATCGTTCCCACTGCGATGCTGGTTGCCAACGATCAGATGGCGCTGGGCGCAATGCGCGCCATTACCGAGTCCGGGCTGCGCGTTGGTGCGGATATCTCGGTAGTGGGATACGACGATACCGAAGACAGCTCATGTTATATCCCGCCGTTAACCACCATCAAACAGGATTTTCGCCTGCTGGGGCAAACCAGCGTGGACCGCTTGCTGCAACTCTCTCAGGGCCAGGCGGTGAAGGGCAATCAGCTGTTGCCCGTCTCACTGGTGAAAAGAAAAACCACCCTGGCGCCCAATACGCAAACCGCCTCTCCCCGCGCGTTGGCCGATTCATTAATGCAGCTGGCACGACAGGTTTCCCGACTGGAAAGCGGGCAGTGAGCGCAACGCAATTAATGTAAGTTAGCTCACTCATTAGGCACCGGGATCTCGACCGATGCCCTTGAGAGCCTTCAACCCAGTCAGCTCCTTCCGGTGGGCGCGGGGCATGACTATCGTCGCCGCACTTATGACTGTCTTCTTTATCATGCAACTCGTAGGACAGGTGCCGGCAGCGCTCTGGGTCATTTTCGGCGAGGACCGCTTTCGCTGGAGCGCGACGATGATCGGCCTGTCGCTTGCGGTATTCGGAATCTTGCACGCCCTCGCTCAAGCCTTCGTCACTGGTCCCGCCACCAAACGTTTCGGCGAGAAGCAGGCCATTATCGCCGGCATGGCGGCCCCACGGGTGCGCATGATCGTGCTCCTGTCGTTGAGGACCCGGCTAGGCTGGCGGGGTTGCCTTACTGGTTAGCAGAATGAATCACCGATACGCGAGCGAACGTGAAGCGACTGCTGCTGCAAAACGTCTGCGACCTGAGCAACAACATGAATGGTCTTCGGTTTCCGTGTTTCGTAAAGTCTGGAAACGCGGAAGTCAGCGCCCTGCACCATTATGTTCCGGATCTGCATCGCAGGATGCTGCTGGCTACCCTGTGGAACACCTACATCTGTATTAACGAAGCGCTGGCATTGACCCTGAGTGATTTTTCTCTGGTCCCGCCGCATCCATACCGCCAGTTGTTTACCCTCACAACGTTCCAGTAACCGGGCATGTTCATCATCAGTAACCCGTATCGTGAGCATCCTCTCTCGTTTCATCGGTATCATTACCCCCATGAACAGAAATCCCCCTTACACGGAGGCATCAGTGACCAAACAGGAAAAAACCGCCCTTAACATGGCCCGCTTTATCAGAAGCCAGACATTAACGCTTCTGGAGAAACTCAACGAGCTGGACGCGGATGAACAGGCAGACATCTGTGAATCGCTTCACGACCACGCTGATGAGCTTTACCGCAGCTGCCTCGCGCGTTTCGGTGATGACGGTGAAAACCTCTGACACATGCAGCTCCCGGAGACGGTCACAGCTTGTCTGTAAGCGGATGCCGGGAGCAGACAAGCCCGTCAGGGCGCGTCAGCGGGTGTTGGCGGGTGTCGGGGCGCAGCCATGACCCAGTCACGTAGCGATAGCGGAGTGTATACTGGCTTAACTATGCGGCATCAGAGCAGATTGTACTGAGAGTGCACCATATATGCGGTGTGAAATACCGCACAGATGCGTAAGGAGAAAATACCGCATCAGGCGCTCTTCCGCTTCCTCGCTCACTGACTCGCTGCGCTCGGTCGTTCGGCTGCGGCGAGCGGTATCAGCTCACTCAAAGGCGGTAATACGGTTATCCACAGAATCAGGGGATAACGCAGGAAAGAACATGTGAGCAAAAGGCCAGCAAAAGGCCAGGAACCGTAAAAAGGCCGCGTTGCTGGCGTTTTTCCATAGGCTCCGCCCCCCTGACGAGCATCACAAAAATCGACGCTCAAGTCAGAGGTGGCGAAACCCGACAGGACTATAAAGATACCAGGCGTTTCCCCCTGGAAGCTCCCTCGTGCGCTCTCCTGTTCCGACCCTGCCGCTTACCGGATACCTGTCCGCCTTTCTCCCTTCGGGAAGCGTGGCGCTTTCTCATAGCTCACGCTGTAGGTATCTCAGTTCGGTGTAGGTCGTTCGCTCCAAGCTGGGCTGTGTGCACGAACCCCCCGTTCAGCCCGACCGCTGCGCCTTATCCGGTAACTATCGTCTTGAGTCCAACCCGGTAAGACACGACTTATCGCCACTGGCAGCAGCCACTGGTAACAGGATTAGCAGAGCGAGGTATGTAGGCGGTGCTACAGAGTTCTTGAAGTGGTGGCCTAACTACGGCTACACTAGAAGGACAGTATTTGGTATCTGCGCTCTGCTGAAGCCAGTTACCTTCGGAAAAAGAGTTGGTAGCTCTTGATCCGGCAAACAAACCACCGCTGGTAGCGGTGGTTTTTTTGTTTGCAAGCAGCAGATTACGCGCAGAAAAAAAGGATCTCAAGAAGATCCTTTGATCTTTTCTACGGGGTCTGACGCTCAGTGGAACGAAAACTCACGTTAAGGGATTTTGGTCATGAGATTATCAAAAAGGATCTTCACCTAGATCCTTTTAAATTAAAAATGAAGTTTTAAATCAATCTAAAGTATATATGAGTAAACTTGGTCTGACAGTTACCAATGCTTAATCAGTGAGGCACCTATCTCAGCGATCTGTCTATTTCGTTCATCCATAGTTGCCTGACTCCCCGTCGTGTAGATAACTACGATACGGGAGGGCTTACCATCTGGCCCCAGTGCTGCAATGATACCGCGAGACCCACGCTCACCGGCTCCAGATTTATCAGCAATAAACCAGCCAGCCGGAAGGGCCGAGCGCAGAAGTGGTCCTGCAACTTTATCCGCCTCCATCCAGTCTATTAATTGTTGCCGGGAAGCTAGAGTAAGTAGTTCGCCAGTTAATAGTTTGCGCAACGTTGTTGCCATTGCTGCAGGCATCGTGGTGTCACGCTCGTCGTTTGGTATGGCTTCATTCAGCTCCGGTTCCCAACGATCAAGGCGAGTTACATGATCCCCCATGTTGTGCAAAAAAGCGGTTAGCTCCTTCGGTCCTCCGATCGTTGTCAGAAGTAAGTTGGCCGCAGTGTTATCACTCATGGTTATGGCAGCACTGCATAATTCTCTTACTGTCATGCCATCCGTAAGATGCTTTTCTGTGACTGGTGAGTACTCAACCAAGTCATTCTGAGAATAGTGTATGCGGCGACCGAGTTGCTCTTGCCCGGCGTCAATACGGGATAATACCGCGCCACATAGCAGAACTTTAAAAGTGCTCATCATTGGAAAACGTTCTTCGGGGCGAAAACTCTCAAGGATCTTACCGCTGTTGAGATCCAGTTCGATGTAACCCACTCGTGCACCCAACTGATCTTCAGCATCTTTTACTTTCACCAGCGTTTCTGGGTGAGCAAAAACAGGAAGGCAAAATGCCGCAAAAAAGGGAATAAGGGCGACACGGAAATGTTGAATACTCATACTCTTCCTTTTTCAATATTATTGAAGCATTTATCAGGGTTATTGTCTCATGAGCGGATACATATTTGAATGTATTTAGAAAAATAAACAAATAGGGGTTCCGCGCACATTTCCCCGAAAAGTGCCACCTGAAATTGTAAACGTTAATATTTTGTTAAAATTCGCGTTAAATTTTTGTTAAATCAGCTCATTTTTTAACCAATAGGCCGAAATCGGCAAAATCCCTTATAAATCAAAAGAATAGACCGAGATAGGGTTGAGTGTTGTTCCAGTTTGGAACAAGAGTCCACTATTAAAGAACGTGGACTCCAACGTCAAAGGGCGAAAAACCGTCTATCAGGGCGATGGCCCACTACGTGAACCATCACCCTAATCAAGTTTTTTGGGGTCGAGGTGCCGTAAAGCACTAAATCGGAACCCTAAAGGGAGCCCCCGATTTAGAGCTTGACGGGGAAAGCCGGCGAACGTGGCGAGAAAGGAAGGGAAGAAAGCGAAAGGAGCGGGCGCTAGGGCGCTGGCAAGTGTAGCGGTCACGCTGCGCGTAACCACCACACCCGCCGCGCTTAATGCGCCGCTACAGGGCGCGTCCCATTCGCCA

**Figure S2. pET-21a(+) plasmid map.**
